# Prospective evaluation of different faecal preservation media for travellers’ diarrhoea diagnostic application with multiplex PCR BioFire FilmArray in resource-limited settings

**DOI:** 10.1099/jmm.0.001954

**Published:** 2025-01-31

**Authors:** R. Toriro, S.D. Woolley, I. Hale, C.J. Bennett, C.J. Phelps, W.D. Nevin, D.S. Burns, T. Edwards, N.J. Beeching, M.K. O’Shea, T.E. Fletcher

**Affiliations:** 1 Department of Clinical Sciences, Liverpool School of Tropical Medicine, Pembroke Place, Liverpool, Merseyside, L3 5QA, UK; 2 Royal Centre for Defence Medicine, Mindelsohn Way, Queen Elizabeth Hospital, Edgbaston, Birmingham, B15 2WB, UK; 3 Centre of Defence Pathology, Royal Centre for Defence Medicine, Queen Elizabeth Hospital Birmingham, Mindelsohn Way, Edgbaston, Birmingham, B15 2WB, UK; 4 3 Medical Regiment, Fulwood Barracks, Preston, Lancashire, PR2 8AA, UK; 5 Centre for Drugs and Diagnostics, Liverpool School of Tropical Medicine, Pembroke Place, Liverpool, Merseyside, L3 5QA, UK; 6 Institute of Immunology and Immunotherapy, College of Medical & Dental Sciences, University of Birmingham, Edgbaston, Birmingham, B15 2TT, UK

**Keywords:** enteropathogen, faecal preservation media, faecal sample, gastrointestinal infection, molecular diagnostics, travellers’ diarrhoea

## Abstract

**Introduction.** Immediate identification of travellers’ diarrhoea-causing pathogens may not be possible in remote settings, but samples can be stored for epidemiological and related research. We collected pilot data to evaluate the utility of three different preservation media for testing stored faecal samples compared to immediate testing of fresh samples using the BioFire^®^ FilmArray^®^ multiplex PCR gastrointestinal panel (bioMérieux).

**Gap statement.** No previous studies have demonstrated the utility of testing faecal samples directly by PCR BioFire^®^ FilmArray^®^ following prolonged storage and transportation in OMNIgene^®^, DNA^™^ shield and FTA^™^ cards.

**Aims.** To evaluate the reliability of OMNIgene^®^, DNA shield^™^ and FTA^™^ card faecal storage and transport media in parallel, compared to initial testing of fresh faeces obtained from the same individuals at the time of presentation with diarrhoea in the field compare the results of faecal samples stored and transported at ambient temperature in OMNIgene^®^, DNA shield^™^ and FTA^™^ cards then tested using PCR BioFire^®^ FilmArray^®^ 6–18 months later with those obtained from fresh faecal samples during a diarrhoea outbreak.

**Methodology.** Fresh faecal samples were obtained from British military personnel who developed diarrhoea during deployment to Kenya between February-April 2022. Unpreserved fresh samples were tested onsite using PCR BioFire^®^ FilmArray^®^ and corresponding samples were stored at ambient temperature in OMNIgene^®^200 (DNAgenotek^®^), DNA/RNA shield DX^™^ (Zymo Research) and Whatman FTA™ Elute cards (GE Healthcare) then repatriated to the UK for direct testing by PCR BioFire^®^ FilmArray^®^, 6-18 months later. The most common enteropathogens evaluated were: *Cryptosporidium* spp., Enteroaggregative *Escherichia coli* (*E. coli*; EAEC), Enteropathogenic *E. coli* (EPEC), Shiga toxin-producing *E. coli* (STEC) and *Campylobacter* spp. Test results for the three storage modalities were compared to the fresh sample tests as a reference standard.

**Results.** Samples from 60 individuals [80% male; median (interquartile range) age 24 (22–28) years] were analysed. Test sensitivity for *Campylobacter* spp. and EAEC was high across all three storage modalities (86.4–100%). OMNIgene^®^200 and DNA/RNA shield^™^ showed significant concordance with the reference standard test for other pathogens, but FTA^™^ Elute card tests had low sensitivity for STEC and poor specificity for *Campylobacter* spp. Agreement between FTA^™^ Elute cards and the reference standard test was low-moderate (kappa coefficient ≤0–0.49) for all enteropathogens.

**Conclusions.** This study demonstrates successful PCR BioFire^®^ FilmArray^®^ utility in testing samples stored in different media and is the first to compare the use of OMNIgene^®^200, DNA/RNA shield^™^ and FTA^™^ Elute cards simultaneously with the results of clinical samples. Stored samples were tested up to 18 months later with significant concordance observed in OMNIgene^®^200 and DNA/RNA shield^™^ compared to reference standard testing. The distorted performance of FTA^™^ Elute card testing requires further optimisation. Testing of samples stored in these media is suitable for research studies, but their applicability with other molecular diagnostic platforms, or clinical diagnostics, requires confirmation.

## Availability of data and materials

A copy of data is available from the corresponding author (R.T.; Romeo.Toriro@lstmed.ac.uk) on reasonable request, provided that this meets local ethical and research governance criteria.

## Introduction

Travellers’ diarrhoea (TD) continues to be a significant global problem that can limit time and opportunity during or after travel, often requiring itinerary changes, specific treatment and occasional hospitalisation [1,2]. It can be caused by a range of enteropathogens alone or in combination [1,3,10]. The predominant enteropathogens vary according to geographic location, and aetiology remains unknown in 40–50% of cases despite microbiological evaluation [8,11]. Most cases are self-limiting and do not require laboratory investigations except when associated with pyrexia, dysentery or when persisting for >7 days, especially in returning travellers. During outbreaks, there is an urgent need to identify the aetiology to inform support and control measures [2,12,14].

Molecular platforms are increasingly used in clinical diagnostic laboratories in addition to, or instead of, conventional microscopic, culture and antigen detection techniques. These include automated platforms enabling simultaneous detection of multiple enteropathogens [14,16], but there is no current consensus on the gold standard multiplex PCR system. Culture-independent molecular platforms provide rapid identification of multiple enteropathogens within a few hours [6,15] and can be used in austere settings where culture methods are not available [17]. Compared to conventional techniques, additional pathogens are often detected using such platforms, but these may be of uncertain clinical significance, and test results need careful interpretation [18].

There are few reports of prospective studies employing quantitative PCR (qPCR) assays to cover all major enteropathogen groups and organisms within a single research design [19]. However, the multiplex PCR BioFire^®^ FilmArray^®^ (FilmArray^®^) gastrointestinal (GI) panel (bioMérieux, Marcy-l'Étoile, France) has been used in conventional and austere diagnostic settings [15,16] and is as accurate for rapid multiple-enteropathogen detection as other molecular platforms [15,20,23]. Faecal samples for FilmArray^®^ testing are typically preserved in Cary Blair transport media and can be stored at room temperature for up to 4 days (15–25 °C) or refrigerated (2–8 °C) for up to 4 days [15]. Consideration should be given to logistic challenges in acquiring laboratory consumables in austere environments such as remote military deployment locations, and latitude should be given for improvisation without impacting scientific rigour. We have previously described testing of unpreserved faecal samples by FilmArray^®^ in an outbreak, where some *Cryptosporidium* spp. FilmArray^®^ positive samples were also found to be positive by microscopy at an independent laboratory, thereby confirming on-site results [24].

For research in resource-limited settings, it may be more convenient to store specimens for transport to a better-equipped diagnostic centre for later analysis. Whatman FTA^™^ Elute cards (FTA^™^ cards) (GE Healthcare, Marlborough, USA) are filter paper-based media which enable prolonged specimen storage and have been found to be a promising tool for enteropathogen detection in settings such as remote military environments [25], and as part of research studies [26,31]. Liquid-based faecal DNA stabilisation media such as the OMNIgene^®^200 (OMNIgene^®^) (DNAgenotek^®^, Ottawa, Canada) and DNA/RNA shield DX^™^ (DNA shield^™^) (Zymo Research, Irvine, CA, USA) have been used in gut microbiome and related studies [25,32, 33]. We are unaware of previous use of OMNIgene^®^ and DNA shield^™^ for travellers’ diarrhoea (TD) field studies, despite their use in gut microbiome investigations, and studies on pathogens causing enteric infections [32,33]. We know from individual manufacturer specifications that both media homogenise and stabilise faecal samples at the point of collection. OMNIgene^®^ is suitable for transportation and storage of stabilised DNA for up to 60 days at ambient temperature, and for up to a year between −20 and −80 °C (DNAgenotek^®^), which renders these media ideal tools for resource-limited setting use in epidemiological studies or clinical trials. Similarly, DNA shield**^™^** preserves nucleic acids from faeces and provides a microbial snapshot of the specimen while stabilising it for safe storage and transportation at ambient temperature. RNA can be retrieved for up to a month at ambient temperature, and DNA for up to 2 years. Samples stored in DNA shield**^™^** can be frozen indefinitely (Zymo Research). There are limited data on the clinical use of these media, and their performance characteristics when used directly with the FilmArray^®^ have not been reported before.

The aims of this study were to:

- evaluate the reliability of OMNIgene^®^, DNA shield^™^ and FTA^™^ card faecal storage and transport systems in parallel, compared to initial testing of fresh faeces obtained from the same individuals at the time of presentation with diarrhoea in the field
- compare the results of faecal samples stored and transported at ambient temperature in OMNIgene^®^, DNA shield^™^ and FTA^™^ cards and tested using the FilmArray^®^ 6–18 months later with those obtained from fresh faecal samples during an outbreak of diarrhoea in rural Kenya [24].

## Methodology

### Study design, settings, ethics, on-site specimen collection and testing

Fresh faecal samples were obtained from British military personnel (*n*=124), who developed diarrhoea during a previously described *Cryptosporidium hominis* outbreak from February to April 2022 [24]. All initial samples were tested onsite (the field test) using the *Conformité Européene in vitro* diagnostic medical devices marked FilmArray^®^ according to the manufacturer’s instructions.

### Rationale for molecular platform choice, sample selection and further testing

We are not aware of any reference or gold standard multiplex PCR for GI molecular diagnostics in austere environments. We selected the FilmArray^®^ as the test platform of choice and reference standard test for this study because of its proven utility in austere environments to improve the diagnosis and management of patients presenting with suspected gastroenteritis [24,34]. Its GI panel combines 22 pathogens (Data Sheet S1, available in the online version of this article) in a single cartridge-based test on a random-access system with a turnaround time of less than 2 h. Study funding limited the number of available FilmArray^®^ GI panels to 240. Therefore, 60/124 (48.4%) corresponding samples from each of the three storage media (total 240 samples) were selected. We based our selection on quota sampling to include a broad range of enteropathogens detected by the reference standard test. The following groups were selected: 5/60 (8.3%) negative samples, 19/60 (31.7%) single enteropathogen samples, 20/60 (33.4%) dual enteropathogen samples and 16/60 (26.7%) multiple enteropathogen samples. We set five positives in the reference standard test as a threshold for selecting the most common enteropathogens to include in the evaluation. These are *Cryptosporidium* spp., enteroaggregative *E. coli* (EAEC), enteropathogenic *E. coli* (EPEC), Shiga toxin-producing *E. coli* (STEC) and *Campylobacter* spp.

### Comparison of the different testing modalities

**Fig. 1.**
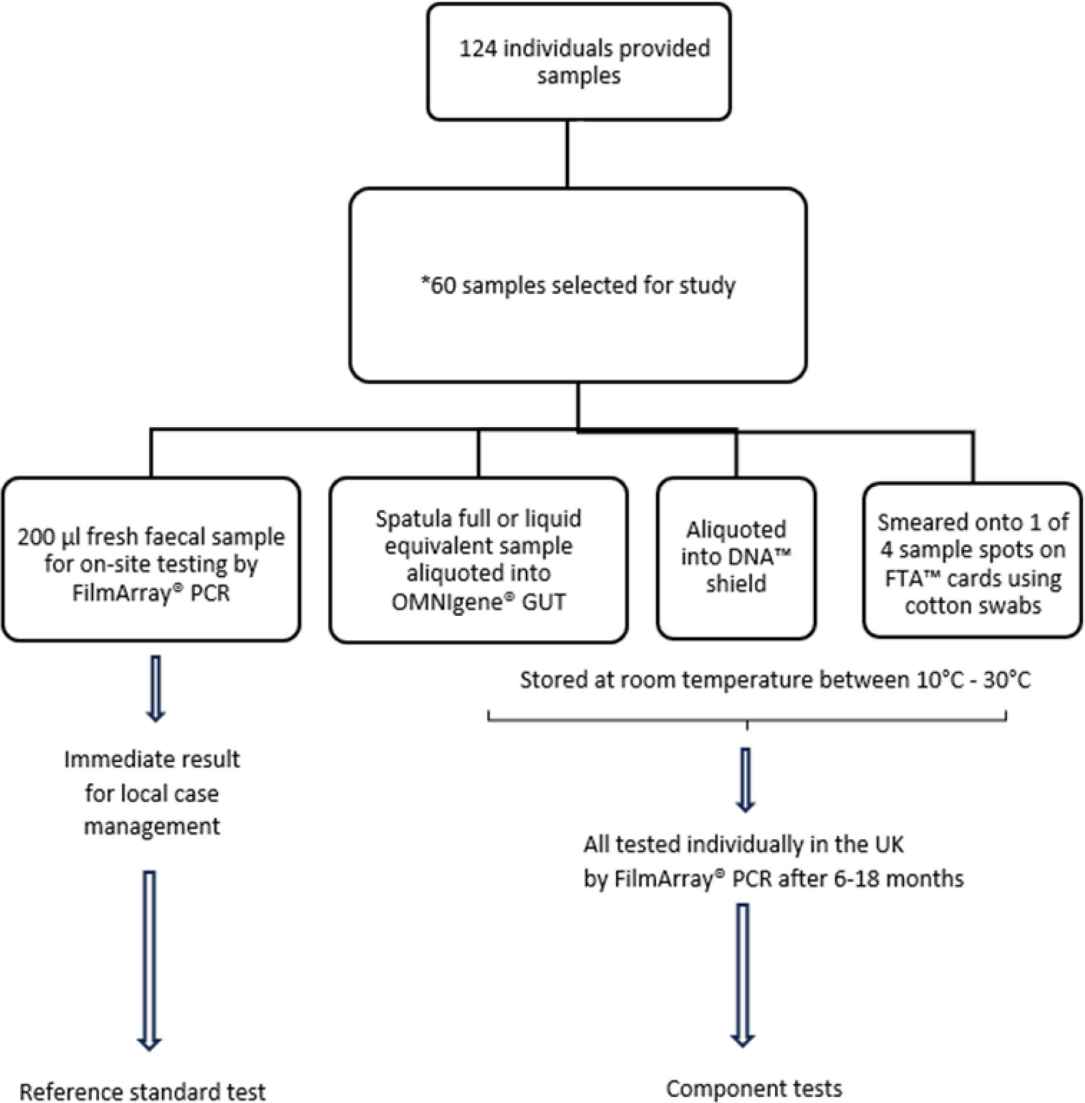
On-site and repatriated faecal sample processing steps. Stepwise approach from onsite sample collection and analysis to repatriation using different storage media, followed by UK laboratory testing at different time points as shown above. *The criteria for the selection of the primary samples have been previously described [60 corresponding samples from each of the three storage media (total 240 samples)].

Individuals self-collected loose faecal samples in 500 ml snap-on lid specimen collection pots before transferring ~10 ml stool volume into 30 ml sterile bottles. Before testing, storage and repatriation, personal identifiers were redacted, and samples marked with unique identifiers were linked to demographic data stored in a multiple-factor authentication encrypted database. Samples were immediately aliquoted without preservatives from their collection pots into the respective storage and transportation media, then shaken vigorously for up to 60 s or until liquified. Samples were not centrifuged and were kept at ambient temperature for further testing in the UK as shown in Fig. 1 [24].

### OMNIgene^®^, DNA shield^™^ and FTA^™^ card (component tests) preparation and testing of repatriated samples

Each of the OMNIgene^®^ and DNA shield^™^ aliquots was shaken vigorously for up to 60 s, or until liquified, and ~0.2 ml homogenized sample was extracted from each using disposable pipettes. The sample was then loaded directly into the sample buffer (FilmArray*^™^* step 2), and the rest of the steps were conducted in accordance with manufacturer guidelines (Data Sheet S2) (bioMérieux).

The manufacturer-validated FTA^™^ card eluate extraction protocol for faeces is described in Data Sheet S3 [35]. Previous use of this and other similar protocols is well-documented [25,27, 29, 36,38], but these methods are protracted and require appropriate expertise and resources which might not be available in austere environments. A customised protocol was developed to streamline the processing steps to allow for direct FTA^™^ card sample testing by FilmArray^™^, with a view to optimizing this for future use in resource-limited environments. We combined this technique into step 2 of the FilmArray^®^ sample processing stage as follows: 1 ml of QIAcard^™^ FTA^™^ wash buffer was transferred into a 1.5 ml Eppendorf Tube^®^. Sample discs measuring 6×2 mm were removed from one of four dried sample spots on which the faecal sample is smeared on the FTA^™^ cards using a Harris Uni-Core disposable punch. DNA/RNA cleaner was used to disinfect and eliminate nucleic acid contaminants on the Harris Uni-Core disposable punch after processing each individual sample. The discs were transferred directly into a wash buffer solution, and the mixture was macerated after which the supernatant was left to sit for 5 min. The 6×2 mm discs were then extracted using a pipette tip and transferred directly into the FilmArray^®^ sample buffer (step 2). The rest of FilmArray^™^ sample testing steps were conducted as per manufacturer guidelines. FTA^™^ cards were kept in separate multi-barrier pouches with desiccant as per the manufacturer’s recommendations (GE Healthcare) to mitigate the risk of cross-contamination.

Negative quality control testing using a blank FilmArray^®^ GI panel was conducted after every ten consecutive tests to rule out cross-contamination. Although investigators were not blind to the reference standard test results, the individual component tests were performed independently, and all corresponding component test results were only plotted against the reference standard test on the conclusion of all testing. Statistical analyses to compare the results were performed thereafter.

### Statistical methods and ethics

The results obtained in the fresh patient sample were taken as the reference standard for comparison with the individual component tests. Sensitivity, specificity, positive predictive value (PPV) and negative predictive value (NPV) were calculated for each storage system, and percentage observed agreement (POA) was used as the final arbiter of agreement between tests. This was calculated as (TP+TN)/(TP+FN+FP+TN)×100, where TP=true positives, TN=true negatives, FN=false negatives and FP=false positives. Invalid or unacceptable results were excluded from the respective totals in each testing modality. Cohen’s kappa (*k*) statistical coefficient was used in Microsoft Excel^®^ (version 2401) to measure the inter-rater reliability and degree of accuracy between tests. Participants provided prior informed written consent for the use of their stored samples in this prospective diagnostic evaluation study. Ministry of Defence Research Ethics Committee (MODREC) ethical approval (2076/MODREC/21) was granted in 2021.

## Results

### Demographics and overall detection rates

Samples were selected from 60 individuals [80% male; median (interquartile range) age 24 (22–28) years] and then matched with corresponding samples in each of the three storage and transport systems.

At least one enteropathogen was detected in 55/60 (91.7%) fresh faecal samples. Results of the component tests were not significantly different: OMNIgene^®^ 54/59 (91.5%) positive with one invalid test; DNA shield^™^ 54/60 (90%) positive; FTA^™^ card 54/58 (93.1%) positive and two invalid results (data not shown).

### Enteropathogens detected by testing modality

The proportion of dual pathogens in the component tests for all pathogens was as follows: OMNIgene^®^ 15/59 (25.4%); DNA shield^™^ 16/60 (26.7%); FTA^™^ card 11/58 (19%). Multiple pathogens were detected in OMNIgene^®^ 15/59 (25%), DNA shield^™^ 13/60 (21.7%) and FTA^™^ card 28/58 (48.3%) (data not shown).

Out of the five selected enteropathogens, *Cryptosporidium* spp. were the most common across all pathogen groups and were mostly detected in the multiple-pathogen samples in the FTA**^™^** card tests [16 (27.6%)]. STEC were frequently detected as a single pathogen, and *Campylobacter* spp. were detected the most as multiple-pathogens in 17 (29.3%) of FTA^™^ card tests compared to only 1 (1.7%) of fresh reference standard tests (Table 1).

**Table 1.** Enteropathogen proportion distribution for each testing modality. The five most common enteropathogens are shown in rows against the four different testing modalities. The corresponding three columns per modality show the number of single, dual and multiple enteropathogens detected alongside each of *Cryptosporidium* spp., EPEC, EAEC, STEC and *Campylobacter* spp

Enteropathogen	Testing modality versus no. of enteropathogens detected*N* (%)											
	Fresh faecal sample*N*=**60**			OMNIgene^®^*N*=**59**			DNA/RNA shield^™^*N*=**60**			FTA^™^ cards*N*=**58**		
	Enteropathogen distribution											
	Single	Dual	Multiple	Single	Dual	Multiple	Single	Dual	Multiple	Single	Dual	Multiple
*Cryptosporidium* spp.	10 (16.7)	6 (10)	9 (15)	12 (20.3)	5 (8.5)	9 (15.3)	12 (60)	4 (6.7)	9 (15)	8 (13.8)	2 (3.4)	16 (27.6)
EPEC	3 (5)	9 (15)	12 (20)	3 (5.1)	11 (18.6)	13 (22)	4(6.7)	9 (15)	10 (16.7)	2(3.4)	4 (6.9)	25 (41.7)
EAEC	0	9 (15)	13 (21.7)	0	7 (11.9)	13 (22)	1 (1.7)	8 (13.3)	11 (18.3)	2 (3.4)	8 (13.8)	26 (44.8)
STEC	3 (5)	5 (8.3)	2 (3.3)	3 (5.1)	0	1 (1.7)	2 (3.3)	1 (1.7)	2 (3.3)	0	0	4 (6.9)
*Campylobacter* spp.	3(5)	2 (3.3)	1 (1.7)	4 (6.8)	1 (1.7)	1 (1.7)	4 (6.7)	2 (3.3)	0	4 (6.9)	3 (5.2)	17 (29.3)

### Component tests versus fresh sample (reference standard) test evaluation

**Fig. 2.**
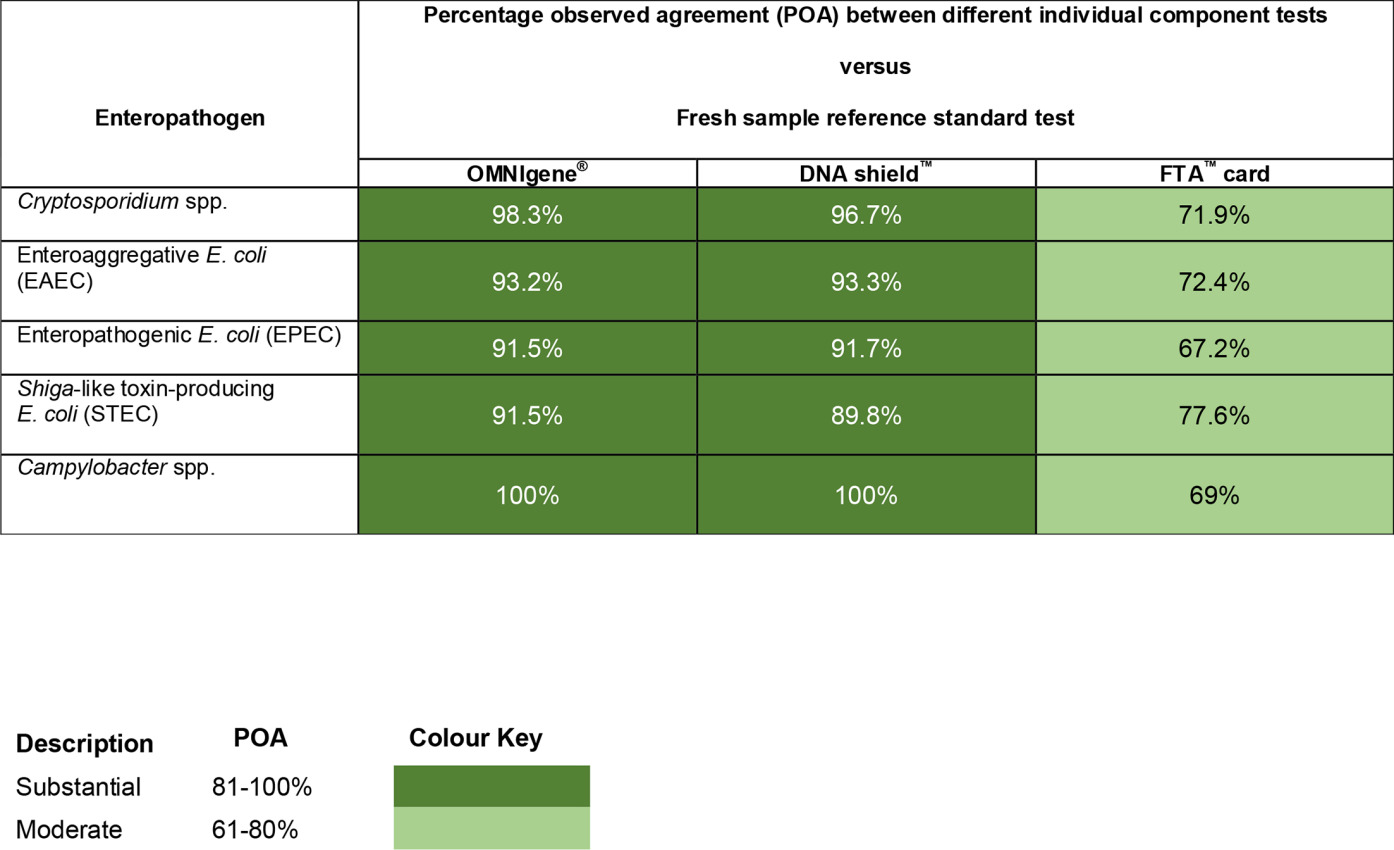
Heatmap summarising differences in percentage observed agreement (POA) between individual tests and reference standard tests.

Compared to fresh sample reference standard testing, each of the three storage modalities had significant sensitivity (70–100%) for all enteropathogens except for STEC. There were no TP results for STEC in the FTA***^™^*** card test, and consequently, sensitivity was poor (0; 95% CI, 0–33.63%) when compared to the reference standard test samples. OMNIgene^®^ and DNA shield**^™^** tests were also highly specific for all enteropathogens (88.6–100%) compared to reference standard sample testing. FTA card^™^ specificity was low for all enteropathogens except EPEC. There was generally very significant concordance in OMNIgene^®^ and DNA shield**^™^** testing for all enteropathogens. However, apart from EPEC, FTA**^™^** card tests only had moderate POAs of between 69 and 77.6% compared to fresh reference standard tests (Tables2^4^, Fig. 2).

**Table 2.** Comparison between the OMNIgene® against the reference standard test

Pathogen	OMNIgene^®^ versus reference standard test									
	TP	TN	FP	FN	Sensitivity (%)95% **CI**	Specificity (%)95% **CI**	PPV (%)95% **CI**	NPV (%)95% **CI**	POA(%)	*k*
*Cryptosporidium* spp.	25	33	1	0	100(86.28–100%)	97.1(84.67–99.93%)	96.2(78.38–99.42%)	100(89.42–100%)	98.3	0.96
EAEC	19	36	1	3	86.4(65.09–97.09%)	97.3(85.84–99.93%)	95(73.18–99.25%)	93.2(80.72–97.17%)	93.2	0.85
EPEC	23	31	4	1	95.8(78.88–99.89%)	88.6(73.26–96.8%)	85.2(69.49–93.56%)	96.9(81.93–99.53%)	91.5	0.83
STEC	4	50	0	5	44.4(13.7–78.8%)	100(92.89–100%)	100(39.76–100%)	90.9(84.79–94.72%)	91.5	0.58
*Campylobacter* spp.	6	53	0	0	100(54.07–100%)	100(93.28–100%)	100(54.07–100%)	100(93.28–100%)	100	1

*k* = Cohen's kappa coefficient with the interpretation of descriptions as below.

≤0 = no agreement.

0.01–0.20 = none to slight.

0.21–0.40 = fair.

0.41– 0.60 = moderate.

0.61–0.80 = substantial.

0.81–1.00 = almost perfect agreement.

FNfalse negativeFPfalse positiveNPVnegative predictive valuePOApercentage observed agreement.PPVpositive predictive valueTNtrue negativeTPtrue positive

**Table 3.** DNA shield^™^ versus reference standard test

Pathogen	DNA shield^™^ versus reference standard test									
	TP	TN	FP	FN	Sensitivity (%)95% **CI**	Specificity (%)95% **CI**	PPV (%)95% **CI**	NPV (%)95% **CI**	POA(%)	*k*
*Cryptosporidium* spp.	24	34	1	1	96(79.65–99.9%)	97.1(85.08–99.93%)	96(77.64–99.40%)	97.14(83.27–99.57%)	96.7	0.93
EAEC	19	37	1	3	86.4(65.09–97.09%)	97.4(86.19–99.93%)	95(73.17–99.25%)	92.5(81.14–97.25%)	93.3	0.85
EPEC	21	34	2	3	87.5(67.64–97.34%)	94.4(81.34–99.32%)	91.3(73.03–97.6%)	91.9(79.68–97.04%)	91.7	0.83
STEC	4	49	1	5	44.4(13.70–78.8%)	98(89.35–99.95%)	80(33.47–96.95%)	90.7(84.51–94.62%)	89.8	0.52
*Campylobacter* spp.	6	54	0	0	100(54.07–100%)	100(93.4–100%)	100(54.07–100%)	100(93.40–100%)	100	1

k = Cohen's kappa coefficient with the interpretation of descriptions as below:

≤0 = no agreement.

0.01–0.20 = none to slight.

0.21–0.40 = fair.

0.41– 0.60 = moderate.

0.61–0.80 = substantial.

0.81–1.00 = almost perfect agreement.

FNfalse negativeFPfalse positiveNCnot computedNPVnegative predictive valuePOApercentage observed agreementPPVpositive predictive valueTNtrue negativeTPtrue positive

**Table 4.** Comparison between the FTA™ card test against the reference standard test

Pathogen	FTA^™^ card versus reference standard test									
	TP	TN	FP	FN	Sensitivity (%)95% **CI**	Specificity (%)95% **CI**	PPV (%)95% **CI**	NPV (%)95%	POA(%)	*k*
*Cryptosporidium* spp.	17	24	9	7	70.8(48.91–87.38%)	72.7(54.48–86.7%)	65.4(50.56–77.72%)	77.4(63.98–86.87%)	71.9	0.43
EAEC	21	21	16	0	100(83.89–100%)	56.8(39.49–72.9%)	56.8(47.57–65.50%)	100(83.89–100%)	72.4	0.49
EPEC	18	21	13	6	75(53.29–90.23%)	61.8(43.56–77.83%)	58.1(46.00–69.23%)	77.8(62.51–88.02%)	67.2	0.35
STEC	0	45	4	9	0(0–33.63%)	91.8(80.4–97.73%)	0	83.3(82.14–84.46%)	77.6	NC
*Campylobacter* spp.	6	34	18	0	100(54.07–100%)	65.4(50.91–78.03%)	25(18.66–32.63%)	100(89.72–100%)	69	0.28

*k* = Cohen's kappa coefficient with the interpretation of descriptions as below:

≤0 = no agreement.

0.01–0.20 = none to slight.

0.21–0.40 = fair.

0.41–0.60 = moderate.

0.61–0.80 = substantial.

0.81–1.00 = almost perfect agreement.

FNfalse negativeFPfalse positiveNPVnegative predictive valuePOApercentage observed agreementPPVpositive predictive valueTNtrue negativeTPtrue positive

## Discussion

We have successfully demonstrated the utility of testing faecal samples preserved in OMNIgene^®^, DNA shield^™^ and FTA^™^ cards by FilmArray^®^ 6–18 months after collection. This is the first report where the FilmArray^®^ has been used directly with samples transported in these storage systems. It is also the first reported parallel comparison of test results in all three storage systems with results of immediate testing of fresh clinical samples. We noted discrepancies with the FilmArray^®^ which include recording a result of ‘not applicable’ for EPEC when STEC is detected because the target (*eae* gene) is present in some STEC strains. A ‘not applicable’ result is also reported for *E. coli* O157 when STEC is not detected; otherwise, the assay may identify *E. coli* O157 strains that lack *stx1* and *stx2* genes [15]. We have shown significant concordance between both the OMNIgene^®^ and DNA shield^™^ results with tests on fresh samples, but the results of tests for enteropathogenic bacteria in FTA^™^ card samples were less consistent. Possible reasons for this are discussed later.

At the time of testing, all OMNIgene^®^ samples had exceeded the manufacturer-recommended storage duration by between 6 and 18 months because of delayed repatriation of samples due to logistic reasons. However, we found no significant degradation of results. We are not aware of any previous successful testing of out-of-date OMNIgene^®^-preserved faecal samples. DNA shield**^™^** and FTA**^™^** cards were tested between 12 and 18 months of collection, which falls within the recommended timelines. We also describe the accurate detection of protozoan parasites in OMNIgene^®^-preserved faecal samples for the first time.

FTA^™^ cards have been used in several other studies testing stored faeces using commercial or bespoke PCR systems [25], and we were able to rule out cross-contamination as a possible cause of increased *Campylobacter* spp. and EAEC detection in FTA**^™^** cards because results from all other component tests for each pathogen were generally concordant, and cross-contamination risk was mitigated in our protocol as described earlier.

### Applicability of testing modalities to diarrhoea diagnostics

In a previous prospective study of TD utilizing qPCR assays to screen for pathogens, diarrhoeagenic *E. coli* were the most prevalent enteropathogens found [19], which is similar to our own findings. This was followed by viral pathogens, in particular, linked to antibiotic use, but parasitic targets were rarely found [19]. Some of the tests that we conducted as part of the wider study, which are not evaluated in detail in this paper, detected single enteropathogens (data not shown).

### Limitations

Multiplex PCR technology has been used in resource-limited environments but is expensive [39,40], and for that reason, testing of samples was conducted at irregular intervals ranging from 6 to 18 months as funding for the procurement of FilmArray^®^ GI panels became available. Logistical challenges were also experienced in the repatriation of the samples resulting in delays. As a result, baseline tests for these media were not conducted, and consequently, time points for loss of enteropathogen integrity cannot be identified. This would have enabled us to separate the impact of the collection device from storage duration on detection, particularly for FTA^TM^ card tests. Nevertheless, due to the limited sample size, our study would, in any event, have been underpowered to assess the impact of storage duration.

Some studies suggest that it is unlikely that real-time field diagnostics could change patient management decisions [2,6], although we previously reported good FilmArray^®^ utility in managing a TD outbreak [24]. The detection of colonisation rather than infection in some cases complicates the interpretation and application of results [41]. Logistical challenges in collecting samples from a highly dispersed and mobile population can result in prolonged storage of faecal samples at ambient temperature, which could inhibit enteropathogen detection. In part, this is due to dietary components frequently found in faecal samples which could interfere with DNA amplification processes leading to FN PCR results [42,43]. Similarly, increased storage duration could lead to degradation of DNA/RNA, or overgrowth by some enteropathogens [44]. The reasons for the distorted performance of tests on samples stored on FTA™ cards are unclear. It is possible that the method that we developed empirically to try and streamline analysis for possible use in a remote setting could have been inadequate for removing contaminants or PCR inhibitors including extracting the DNA/RNA bound in the matrix, which might impact on result accuracy. Due to limited resources, we could only conduct quota sampling of 60/124 (48.4%) samples from each storage modality, and we were unable to compare results for the same stored samples at different time points.

### Significance of findings

We have shown that individually, tests on faecal samples stored in DNA shield**^™^** or OMNIgene^®^ systems demonstrate excellent concordance with fresh faecal sample testing by FilmArray**^™^**. These systems demonstrate potential for faecal storage in resource-limited settings where FilmArray^™^ capability is not readily available. Apart from the use of real as opposed to spiked faecal samples, one strength of this study was not adding preservatives to samples placed in cold storage due to the likelihood of interference with PCR amplification processes, particularly after extended storage [45,46]. We have also shown that storage duration in tropical ambient temperature conditions had minimal impact on pathogen recovery as demonstrated by the general concordance of OMNIgene^®^ and DNA shield**^™^** versus the fresh sample reference standard test results. FTA**^™^** cards are compact and durable once secured in their storage pouches, but further work is required to optimise their use with the FilmArray**^™^**. DNA shield**^™^** and OMNIgene^®^ are potentially more unwieldy and fragile for use in the field, and, perhaps, studies should be considered to assess their acceptability and robustness when used by individual travellers or military personnel.

### Suggested future studies

Prospective observational studies should evaluate the impact of storage duration on pathogen recovery at different time points. Further work is also needed to determine whether reagents used with or in the different storage systems interfere with faecal PCR inhibitors or otherwise underline the discrepancies that we observed with FTA**^™^** cards. Other molecular platforms should be evaluated using faecal samples stored in the different systems, compared to testing fresh samples and samples frozen and stored without preservatives. User-oriented studies should evaluate the suitability of different storage systems for use directly by travellers rather than in a laboratory setting.

## Conclusions

Our pilot data suggest that diarrhoeal samples can be stored in OMNIgene^®^ and DNA shield^™^ for up to 18 months at ambient temperature without significant impact on their performance characteristics when tested for three common enteropathogens directly by PCR FilmArray^™^. These media could be suitable for epidemiological studies. However, additional studies are needed to improve on our customised FTA^™^ card protocol followed by a more comprehensive evaluation of all three media in parallel across different enteropathogens and time points.

## supplementary material

10.1099/jmm.0.001954 Uncited Supplementary Data Sheet 1.

## Abbreviations

EAEC: enteroaggregative *Escherichia coli*
EPEC: enteropathogenic *Escherichia coli*
FN: false negative
FP: false positive
GI: gastrointestinal
MODREC: Ministry of Defence Research Ethics Committee
NPV: negative predictive value
POA: percentage observed agreement
PPV: positive predictive value
qPCR: quantitative PCR
STEC: Shiga toxin-producing *Escherichia coli*
TD: travellers’ diarrhoea
TN: true negative
TP: true positive
