# Supplementary material for: Prospective evaluation of different faecal preservation media for travellers’ diarrhoea diagnostic application with multiplex PCR BioFire FilmArray in resource-limited settings
Source: J Med Microbiol. 2025 Jan 31;74(1):001954. doi: 10.1099/jmm.0.001954 (PMC11784588; doi:10.1099/jmm.0.001954)
Supplement: Uncited Supplementary Data Sheet 1. [file jmm-74-01954-s001.pdf]

Supplementary Data Sheet 1. PCR BioFire® FilmArray® GI Panel targets

The 22 enteropathogen targets for PCR BioFire® FilmArray® directly from faecal specimens

Bacteria

- *Campylobacter* (*jejuni, coli* & *upsaliensis*)
- *Clostridium difficile* (Toxin A/B)
- *Plesiomonas shigelloides*
- *Salmonella spp.*
- *Yersinia enterocolitica*
- *Vibrio* (*parahaemolyticus, vulnificus*, & *cholerae*)
- *Vibrio cholerae*

Diarrhoeagenic *E. coli or Shigella spp.*

- *E. coli* O157
- Enteroaggregative *E. coli* (EAEC)
- Enteroinvasive *E. coli* (EIEC)
- Enteropathogenic *E.* coli (EPEC)
- Enterotoxigenic *E. coli* (ETEC) lt/st
- Shiga-like toxin-producing *E. coli* (STEC) stx1/stx2 *E. coli* O157
-

Viruses

- Adenovirus F 40/41
- Astrovirus
- Norovirus GI/GII
- Rotavirus A
- Sapovirus (I, II, IV, and V)

Parasites

- *Cryptosporidium*
- *Cyclospora cayetanensis*
- *Entamoeba histolytica*
- *Giardia lamblia*

Reference:

Buss SN, Leber A, Chapin K, et al. Multicenter evaluation of the BioFire® FilmArray® Gastrointestinal Panel for etiologic diagnosis of infectious gastroenteritis. J Clin Microbiol 2015; 53:915–925. doi:10.1128/JCM.02674-14.

Supplementary Data Sheet 2: BioFire™ FilmArray™ PCR testing protocol

1. Place the FilmArray® pouch into the loading block and inject the hydration solution into the pouch through the blue inlet port on the right.
2. Add approximately 0.2ml of sample into the sample buffer, mix the solution then inject it into the pouch through the red inlet port on the left.
3. Load the FilmArray® pouch into the FilmArray® instrument and scan the pouch ID using the barcode reader.
4. Finally, enter any unique sample identifiers and start the automated run which completes by producing a test report.

(bioMérieux, Marcy-l'Étoile, France).

Supplementary Data Sheet 3: DNA isolation using QIAamp DNA Investigator Kit

This kit is provided with reagents and columns.

1. Use a Harris Uni-Core disposable punch (1.2, 2.0, 3.0, or 6.0 mm) to remove a sample disc from the centre of a dried sample spot. Place the disc in a clean RNase/DNase-free 1.5 ml microcentrifuge tube.
2. Add 280 µl of ATL buffer to the tube.
3. Add 20 µl of Proteinase K and vortex for 30 sec.
4. Heat for 60 min at 56°C, vortexing for 30 sec every 10 min.
5. Centrifuge at maximum speed in a microcentrifuge for 30 sec.
6. Add 300 µl of AL buffer and vortex for 10 sec.
7. Incubate at 70°C for 10 min, vortexing for 10 sec every 3 min.
8. Centrifuge at maximum speed for 30 sec and transfer the lysate to a min Elute column.
9. Centrifuge at 6000 × g for 1 min and discard the flowthrough.

(GE Healthcare, Europe, GmbH, Germany)
